# Glial hypothalamic inhibition of GLUT2 expression alters satiety, impacting eating behavior

**DOI:** 10.1002/glia.23267

**Published:** 2017-11-27

**Authors:** María J. Barahona, Paula Llanos, Antonia Recabal, Kathleen Escobar‐Acuña, Roberto Elizondo‐Vega, Magdiel Salgado, Patricio Ordenes, Elena Uribe, Fernando J. Sepúlveda, Ricardo C. Araneda, María A. García‐Robles

**Affiliations:** ^1^ Laboratorio de Biología Celular, Departamento de Biología Celular Facultad de Ciencias Biológicas, Universidad de Concepción Concepción Chile; ^2^ Departamento de Bioquímica y Biología Molecular Universidad de Concepción Chile; ^3^ Departamento de Ciencias Biológica Universidad Andrés Bello Concepción Chile; ^4^ Department of Biology University of Maryland College Park Maryland; ^5^ Laboratorio de Inmunología Celular y Molecular, Centro de Investigación Biomédica, Facultad de Medicina, Universidad de los Andes Santiago Chile

**Keywords:** glucosensing, GLUT2, hypothalamus, knockdown, tanycytes

## Abstract

Glucose is a key modulator of feeding behavior. By acting in peripheral tissues and in the central nervous system, it directly controls the secretion of hormones and neuropeptides and modulates the activity of the autonomic nervous system. GLUT2 is required for several glucoregulatory responses in the brain, including feeding behavior, and is localized in the hypothalamus and brainstem, which are the main centers that control this behavior. In the hypothalamus, GLUT2 has been detected in glial cells, known as tanycytes, which line the basal walls of the third ventricle (3V). This study aimed to clarify the role of GLUT2 expression in tanycytes in feeding behavior using 3V injections of an adenovirus encoding a shRNA against GLUT2 and the reporter EGFP (Ad‐shGLUT2). Efficient *in vivo* GLUT2 knockdown in rat hypothalamic tissue was demonstrated by qPCR and Western blot analyses. Specificity of cell transduction in the hypothalamus and brainstem was evaluated by EGFP‐fluorescence and immunohistochemistry, which showed EGFP expression specifically in ependymal cells, including tanycytes. The altered mRNA levels of both orexigenic and anorexigenic neuropeptides suggested a loss of response to increased glucose in the 3V. Feeding behavior analysis in the fasting‐feeding transition revealed that GLUT2‐knockdown rats had increased food intake and body weight, suggesting an inhibitory effect on satiety. Taken together, suppression of GLUT2 expression in tanycytes disrupted the hypothalamic glucosensing mechanism, which altered the feeding behavior.

## 1 INTRODUCTION

GLUT2 is a member of the facilitated glucose transporter family (GLUT), which is encoded by the *SLC2A* gene family. Due to its kinetic properties and tissue localization, GLUT2 is involved in the glucosensing mechanism as it has a uniquely low affinity for glucose (Km ∼17 mM) and can also use mannose, galactose, and fructose as low affinity substrates for transport (Thorens, Guillam, Beermann, Burcelin, & Jaquet, 2000). GLUT2 is the major glucose transporter present in hepatocytes, enterocytes, kidney epithelial cells, and cells of the hepatoportal vein (Thorens, 2015). GLUT2 is also the major glucose transporter in pancreatic ß‐cells, where its genetic inactivation impairs glucose uptake and suppresses glucose‐stimulated insulin secretion. GLUT2^−/−^ mice die at around the weaning period, and transgenic expression of another glucose transporter, GLUT1, in β‐cells (RIPGlut1;GLUT2^−/−^) restores normal glucose‐stimulated insulin biosynthesis (Bady et al., 2006; Guillam et al., 1997; Thorens et al., 2000).

In the central nervous system, GLUT2 immunohistochemical studies are limited by its low level of expression (Arluison, Quignon, Nguyen, et al., 2004; Garcia et al., 2003; Maekawa et al., 2000). However, those that exist have been corroborated by the use of mice expressing a fluorescent reporter gene (eYFP) under the control of the GLUT2 promoter, GLUT2‐eYFP mice (Mounien et al., 2010). GLUT2 was found in neurons and astrocytes dispersed in many structures, including the hypothalamus, the brain stem, the thalamic area (Arluison, Quignon, Thorens, Leloup, & Penicaud, 2004; Labouebe, Boutrel, Tarussio, & Thorens, 2016) and in tanycytes (Garcia et al., 2003). Tanycytes are radial glial‐like cells surrounding the lateral walls of the infundibular recess (Recabal, Caprile, & Garcia‐Robles, 2017). Their apical poles contact the cerebrospinal fluid (CSF), and basal extensions project into the arcuate nucleus (AN) (Flament‐Durand & Brion, 1985). Tanycytes are classified into four main groups on the basis of differences in their anatomical localization and gene expression: α1, α2 (Robins et al., 2013), β1, and β2 (Elizondo‐Vega et al., 2015; Langlet, Mullier, Bouret, Prevot, & Dehouck, 2013). β2‐tanycytes cover the floor of the 3V; in their apical face, they present tight junctions that form the CSF‐median eminence (ME) barrier and extend their projections inside the ME. Interestingly, these tight junctions and cellular contacts can change, depending on the metabolic state of the organism (Langlet et al., 2013). Furthermore, GLUT2‐positive α2‐ and β1‐tanycytes are located in the lateral walls of the 3V and make contact with orexigenic AN neurons, which produce neuropeptide Y (NPY) and agouti‐related protein (AGRP), and anorexigenic AN neurons, which produce proopiomelanocortin (POMC) and the cocaine‐amphetamine‐regulated transcript (CART), through their extensive processes (Broberger, Johansen, Johansson, Schalling, & Hokfelt, 1998; Elias et al., 1998; Kristensen et al., 1998). Interestingly, GLUT2‐eYFP mice showed the absence of labeling in POMC or NPY neurons (Mounien et al., 2010); however, these mice showed labeled nerve terminals, presumably from GLUT2‐expressing cells, which have their soma outside the AN, suggesting an indirect control of AN neurons by glucose (Mounien et al., 2010; Thorens, 2005). Recently, GLUT2 was also detected in neurons of the nucleus tractus solitarius (NTS), specifically in a hypoglycemia‐activated neuronal population, which stimulates vagal activity and glucagon secretion, indicating a role for GLUT2 in the hypoglycemic condition (Lamy et al., 2014).

Several studies support a role for GLUT2 in feeding behavior. Specifically, central administration of 2‐deoxyglucose (2‐DOG), a nonmetabolic substrate of GLUT, induced food intake and increased the expression of orexigenic neuropeptides in the AN (Miselis & Epstein, 1975). Interestingly, ripglut1; GLUT2^−/−^ mice exhibit increased food intake in the fasting‐feeding transition and deregulated orexigenic and anorexigenic neuropeptide expression in response to intracerebroventricular (icv) glucose (Bady et al., 2006). In contrast, icv injections of antisense constructs, designed to specifically to silence GLUT2 expression, reduced feeding and body weight gain in rats (Leloup, Orosco, Serradas, Nicolaidis, & Penicaud, 1998). In mice with specific inactivation of GLUT2 in the central and peripheral nervous systems, no differences in body weight were observed, but progressive glucose intolerance developed (Tarussio et al., 2014).

Although the expression of several proteins involved in the detection and response to glucose has been demonstrated in tanycytes (Cortes‐Campos et al., 2011; Garcia et al., 2003; Millan et al., 2010), there was no evidence of their involvement in the regulation of food intake. We used a molecular tool for *in vivo* GLUT2 knockdown in specific cells of the rat brain. Our results showed that 3V injection of an adenovirus encoding a shRNA against GLUT2 and the reporter enhanced green fluorescent protein (EGFP), Ad‐shGLUT2, efficiently transduced tanycytes and ependymal cells of the 3V and fourth ventricle (4V) without affecting neurons or astrocytes. GLUT2‐knockdown failed to exhibit the normal response to glucose, a decrease in orexigenic neuropeptides and an increase in anorexigenic neuropeptide. Moreover, feeding behavior analysis showed that GLUT2 inhibition produces alterations in feeding macro and microstructure‐related parameters. Thus, an increase in food intake, body weight and consumption rate was observed, as well as a decrease in the duration of meal intervals.

## 2 MATERIALS AND METHODS

### 2.1 Ethics statement

All **s**tudies were reviewed and approved by the Animal Ethics Committee of the Chile's National Commission for Scientific and Technological Research (CONICYT, protocol for projects # 1140677). All animal work was approved by the appropriate Ethics and Animal Care and Use Committee of the Universidad de Concepcion, Chile. Animals were treated in compliance with the U.S. National Institutes of Health guidelines for animal care and use. Male adult Sprague‐Dawley rats (200–280 g) were housed in a 12‐h light/dark cycle with food and water *ad libitum*. Animals were fed a standard chow diet (Lab Diet, ProLab) containing no less than 5% crude fat. Feeding behavior analysis was performed in a stress‐free condition.

### 2.2 Generation of adenoviral shRNA‐GLUT2 vectors

Oligonucleotides targeting rat GLUT2 were designed and selected using the NCBI sequence, NM_012879.2. Sense siRNA‐rGLUT2 5′‐CGC GCC GCC TGG ATG ACC GAA GAG CTA TTC AAG AGA TAG CTC TTC GGT CAT CCA GTT TTT TTA AT ‐3′ and antisense siRNA‐rGLUT2 5′‐GGC GGA CCT ACT GGC TTC TCG ATA AGT TCT CTA TCG AGA AGC CAG TAG GTC AAA AAA AAT‐3′ shared no homology with other rat coding sequences by BLAST analysis. A ring sequence of nine base pairs (TTC AAG AGA) existed between the sense and antisense strands. Control shRNAs were designed and selected to target β‐galactosidase from *E*. *coli*: sense shRNA‐βGal 5′‐CGC GCC AAG GCC AGA CGC GAA TTA TTT CAA GAG AAT AAT TCG CGT CTG GCC TTT TTT TTT TAA T‐3′ and antisense siRNA‐β Gal: 5′‐TAA AAA AAA AAG GCC AGA CGC GAA TTA TTC TCT TGA AAT AAT TCG CGT CTG GCC TTG G‐3′. All shRNAs were synthesized by Integrated DNA Technologies (Coralville, IA, USA) and designed to contain both AscI and PacI restriction enzyme sites, which were used for ligation into pDC311.2‐OFF‐EGFP downstream of the human H1 promoter. The plasmid was then cotransfected with an Ad genomic plasmid, pBHGloxΔE1,3Cre (Admax system, Microbix Biosystems Inc., Mississauga, Ontario, Canada) into HEK293A cells. Virus particles were released by heat shock, and cell debris was removed by centrifugation for 5 min at 5,000g. The particles were recovered from the supernatant by filtration through a 0.45‐µm filter.

### 2.3 Primary culture of tanycytes

Hypothalamic glial cell cultures from 1‐day postnatal brain samples were prepared following the method described previously (Cortes‐Campos et al., 2011; Garcia et al., 2005; Orellana et al., 2012) Briefly, the hypothalamic region was removed from the brain and further dissected to obtain the tissue containing the ependymal layer. Samples were incubated with 0.25% trypsin‐0.2% EDTA (w/v) for 20 min at 37°C. Cells were seeded at a density of 1.2 × 10^5^ cells/cm^2^ in MEM supplemented with 10% FBS, 2 mM L‐glutamine, 100 U/ml penicillin, 100 mg/ml streptomycin, and 2.5 mg/ml fungizone (Thermo Fisher Scientific Inc.) at 37°C and 5% CO_2_ in a humidified atmosphere. Dishes with the highest density of confluent epithelial cells were expanded for subsequent adenoviral transduction to measure cell survival and transduction efficiency. Cells were grown on poly‐L‐lysine‐coated glass cover slides in 24‐well plates in serum‐free MEM, and then cells were infected with serotype 5 adenovirus Ad‐GLUT2‐shRNA or Ad‐βgal‐shRNA (control) at 5 × 10^6^ ifu/ml. The virus‐containing medium was replaced after 24 hr with MEM containing 10% (v/v) FBS and incubated for 48, 72 and 96 hr. Transduction efficiency was calculated as the percentage of total cells obtained using the nuclear marker, TOPRO‐3 (1:1,000, Invitrogen), which were also EGFP‐positive. Slides were analyzed using confocal laser microscopy (Zeiss LSM700). Tanycytes were grown in 6‐well and 12‐well plates in serum‐free media and infected with serotype 5 adenovirus Ad‐GLUT2‐shRNA or Ad‐βgal‐shRNA at 5 × 10^6^ ifu/ml. The virus‐containing medium was replaced after 24 hr with MEM containing 15 mM glucose and 10% (v/v) FBS and incubated for a total of 48 hr at 37°C and 5% CO_2_ in a humidified atmosphere. mRNA and protein levels were quantified as described below. For detecting lost of function, uptake assays were performed at 5 min, at 37°C in 0.5 ml of incubation buffer containing 20 mM 2‐DOG and 10 µCi of 2‐deoxy‐D‐[1,2‐(N)3H]glucose (30.6 Ci/mmol; DuPont–NEN, Boston, MA, USA).

### 2.4 Icv injections of ad‐shGLUT2 and ad‐shβgal

Rats were anesthetized with an intraperitoneal injection mix of ketamine‐xilazine (90 mg/kg‐10 mg/kg). Using a cannula (28 gauge), 20 μL of adenovirus (2 × 10^9^ ifu/ml) was injected (2.5 µL/min) into the 3V using a stereotactic apparatus (AP −3.14 mm, ML 0.0 mm, DV 9.2 mm). Samples of the hypothalamus were collected after 48 hr for subsequent extraction of protein and RNA as well as immunohistochemistry (further described below). For feeding and neuropeptide analyses, stainless steel cannulas were stereotactically implanted in the 3V of rats and secured to the skull with dental acrylic. Rats were housed alone following surgery and allowed to recover for 5 days before adenovirus administration.

### 2.5 Immunoblotting

Total protein extracts were obtained from primary tanycyte cultures and rat hypothalamic samples. Samples were homogenized in buffer A (0.3 mM sucrose, 3 mM DTT, 1 mM EDTA, 100 µg/ml PMSF, 2 µg/ml pepstatin A, 2 µg/ml leucopeptin, and 2 mg/ml aprotinin), sonicated three times on ice at 60 Hz for 10 s (Sonics & Material INC, VCF1, Newtown, CT, USA), and separated by centrifugation at 8,000g for 10 min. Proteins were resolved by SDS‐PAGE (50 µg/lane) in a 12% (w/v) polyacrylamide gel, transferred to PVDF membranes (0.45 mm pore, Amersham Pharmacia Biotech., Piscataway, NJ, USA), and probed for 16 hr at 4°C with rabbit anti‐GLUT2 (1:1,000; Alpha Diagnostic, San Antonio, TX, USA), anti‐GLUT1 (1:1,000; Alomone Labs, Jerusalem, Israel), anti‐glucokinase (GK; 1:2,000; Santa Cruz Biotechnology, Santa Cruz, CA, USA), and anti‐β‐actin (1:10,000; Santa Cruz). After extensive washing, PVDF membranes were incubated for 2 hr with peroxidase‐labeled anti‐rabbit IgG (1:7,000; Jackson ImmunoResearch, West Grove, PA, USA). The reaction was developed using the enhanced chemiluminescence (ECL) Western blot analysis system (Amersham Biosciences, Pittsburgh, PA, USA). Images shown are representative of at least three analyses performed on samples from at least three separate experiments. β‐actin expression levels were used as a loading control for densitometric analysis.

### 2.6 Quantitative reverse transcription‐polymerase chain reaction (qRT‐PCR)

The brain of each rat was removed, and hypothalamic areas ware isolated and further dissected. Total RNA from the hypothalamic samples was isolated using TRIzol (Invitrogen) and treated with DNase I (Fermentas International, Burlington, Ontario, Canada). RT‐PCR was performed according to the manufacturer's protocol (Fermentas International) using 2 µg of RNA. Parallel reactions were performed in the absence of reverse transcriptase to control for the presence of genomic DNA. qRT‐PCR reactions were prepared with a Brilliant II SYBR Green qPCR Master Mix kit (Agilent Technologies, Santa Clara, CA, USA) in a final volume of 20 µL containing 2 µL cDNA and the following sets of primers (500 nM each): GLUT2, sense 5′‐GGC TAA TTT CAG GAC TGG TT‐3′ and antisense 5′‐TTT CTT TGC CCT GAC TTC CT‐3′; NPY, sense 5′‐TGT TTG GGC ATT CTG GCT GAG G‐3′and antisense 5′‐CTG GGG GCA TTT TCT GTG CTT TC‐3′; AGRP, sense 5′‐GCA GAC CGA GCA GAA GAT GTT C‐3′ and antisense 5′‐GTA GCA CGT CTT GAA GAA GC GG‐3′; POMC, sense 5′‐CTC CTG CTT CAG ACC TCC ATA GAC‐3′ and antisense 5′‐AAG GGC TGT TCA TCT CCG TTG‐3′; CART, sense 5′‐TCT GGG AAG AAG AGG GAC TTT CGC‐3′ and antisense 5′‐TCC ATT TGT GTT GCT TTG GG GTG‐3′; cyclophilin, sense 5′‐ATA ATG GCA CTG GTG GCA AGT C‐3′ and antisense 5′‐ATT CCT GGA CCC AAA ACG CTC C‐3′; GK, sense 5′‐AAA GAT GTT GCC CAC CTA CGT GCG‐3′ and antisense 5′‐ATC ATG CCG ACC TCA CAT TGG C‐3′ and GLUT1 5′‐CAT GTA TGT GGG GGA GGT GT‐3′ and antisense 5′‐GAC GAA CAG CGA CAC CAC AG‐3′. Each reaction mixture was incubated at 95°C for 5 min followed by 40 cycles of 30 s at 95°C, 30 s at 55°C, and 30 s at 72°C and a final extension of 7 min at 72°C. The relative expression of GLUT2, GLUT1, GK and neuropeptides was calculated by the comparative CT method using cyclophilin as the housekeeping control gene.

### 2.7 Immunocytochemistry

Animals were injected with the adenovirus as described above, and brains were collected for immunohistochemistry 48 hr later. Rat brains were fixed for 24 hr in 4% paraformaldehyde (PFA), and thick frontal sections of the hypothalamus and brainstem (40 µm) and other brain regions were cut with a cryostat. Tissues were stained with chicken anti‐vimentin (1:200; Millipore, Billerica, MA, USA), rabbit anti‐NeuN (1:5,000; Abcam, Cambridge, MA, USA), mouse anti‐GFAP (1:200; Millipore), and mouse anti‐HuC (1:200; Invitrogen, Rockville, MD, USA). The antibody was diluted in Tris‐HCl buffer (pH 7.8) containing 8.4 mM Na_3_PO_4_, 3.5 mM KH_2_PO_4_, 120 mM NaCl, and 1% bovine serum albumin. Sections were incubated with the antibodies overnight at room temperature in a humid chamber. After washing, sections were incubated for 2 hr at room temperature with Cy2‐ (1:200) Cy3‐ (1:200) and Cy5‐ (1:200; Jackson ImmunoResearch Laboratories) labeled secondary antibodies. These samples were then counterstained with the DNA stain, HOECHST (1:1,000; Invitrogen). The slides were analyzed using confocal‐spectral laser microscopy (LSM 780 NLO, Zeiss).

### 2.8 Food intake monitoring

Prior to adenovirus injection, rats were handled daily for one week to become acclimated to the researchers and experimental procedures. After adenovirus injection, rats were subjected to 24‐h fasting followed by a 24‐h refeeding period. This cycle includes removal of the rat from the cage to measure their food intake and body weight. Food intake was monitored by providing preweighed food over a defined time interval. Food intake was expressed as the amount (in grams) consumed in 24 hr (g/24 hr). Every interaction with the feeder was recorded by a computerized data acquisition system (VitalView, Respironics, Inc, Murraysville, PA, USA), which individually registers the number of times each rat interacted with the feeder and the amount of time that they remained in the trough. A Meal Event (ME) was defined as one or more episodes longer than 5 sec and no longer than 15 min, followed by a meal interval (MI). The minimum MI was defined as 10 min, as previously described (Elizondo‐Vega et al., 2016). When feeding episodes were longer than 30 min, they were considered a new meal. The meal pattern parameters were calculated as follows: meal events (number), meal interval (min), mean meal size (mg/meal), mean meal duration (min/meal), and eating rate (mg/min). The meal intervals were calculated as a period between the end of one meal and the initiation of the next. Meal events were defined as the total meals in 24 hr. The mean meal size was determined as the total food intake (g) divided by frequency. The mean meal duration was calculated by dividing the total meal duration (min) by total meal events, and the eating rate was estimated by dividing total food intake (g) by total meal duration (min).

### 2.9 Statistical analyses

For evaluating qRT‐PCR assays, differences between two groups were assessed using the Student *t*‐test. Differences between groups in feeding behavior assays were assessed using ANOVA. The statistical analyses were performed using GraphPad Prism 5.0 Software (GraphPad Software Inc., San Diego, CA, USA). Results were expressed as mean ± standard deviation (SD).

## 3 RESULTS

### 3.1 Generation of ad‐shGLUT2 and its evaluation in vitro

To examine the role of GLUT2 on brain glucosensing, we used a targeted a knockdown approach using adenovirus constructs. To this extent, we generated two serotype 5 replication‐deficient types of viral particles, Ad‐shGLUT2 and Ad‐shßgal, which were designed to inhibit the expression of GLUT2 and ß‐galactosidase, respectively. In addition, both constructs contained a sequence for EGFP as a reporter gene (Supporting Information Figure S1A). The expression of the EGFP reporter was used to examine the transduction capacity and effect on cell viability of each at 48, 72 and 96 hr (Supporting Information Figure S1B–G) in primary tanycyte cultures. At 48 hr postinfection, the transduction efficiency was approximately 100% with approximately 60% survival for both viruses (Supporting Information Figure S1H,I). At 72 and 96 hr postinfection, the transduction efficiency was reduced, and the viability was decreased at 96 hr post‐transduction. We selected 48 hr postinfection in primary tanycyte cultures to evaluate the inhibitory capacity of Ad‐shGLUT2. qRT‐PCR analysis revealed GLUT2 mRNA was decreased by 75% (Supporting Information Figure S1J). Similarly, comparison of protein extracts obtained from cells transduced with Ad‐shβgal (Supporting Information Figure S1B, lane 1) versus those transduced with Ad‐shGLUT2 (Supporting Information Figure S1B, lane 2) indicated that GLUT2 levels were reduced by 60%. We also evaluated whether GK expression, which is also present in tanycytes (Millan et al., 2010), was altered by GLUT2 inhibition. We detected no significant differences in protein levels between both animal groups, demonstrating the specificity of the GLUT2 shRNA (Supporting Information Figure S1K). Similarly, GLUT1, which is also expressed in tanycytes (Garcia et al., 2001), was not affected by GLUT2 inhibition. EGFP was detected at similar levels in rats transduced with both adenoviruses. The intensity of the bands was normalized to β‐actin and expressed as a percentage of the ratio obtained with the control. Thus, only GLUT2 expression was significantly decreased following injection of the adenovirus carrying the shGLUT2‐RNA, and no compensatory effects on GLUT1 or GK expression occurred under these conditions.

### 3.2 In vivo ad‐shGLUT2 transduction

**Figure 1:**
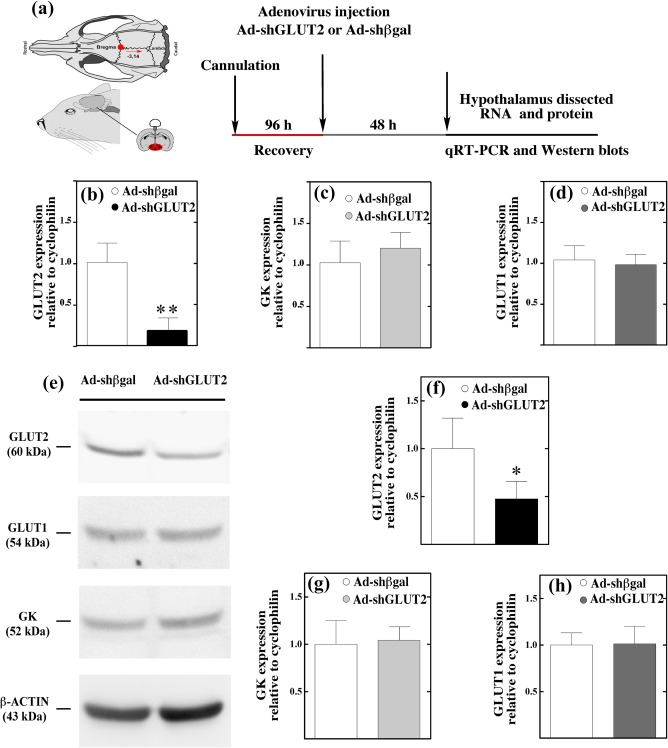
Injection of Ad‐shGLUT2 into the 3V inhibits GLUT2 without altering GK and GLUT1. (a) Experimental protocol. (b‐d) Analysis of GLUT2, GK, and GLUT1 mRNA expression relative to cyclophilin in hypothalamic samples from rats transduced with Ad‐shβgal or Ad‐shGLUT2 by qRT‐PCR. (e) Western blot analysis of hypothalamic protein extracts isolated from rats transduced with Ad‐shβgal (lane 1) or Ad‐shGLUT2 (lane 2) for 48 hr. (f‐h) Densitometric analysis of each protein relative to β‐actin evaluated in Ad‐shGLUT2‐injected rats, as a percentage of levels observed in Ad‐shβgal‐EGFP‐injected rats. Results are representative of four independent experiments performed in triplicate. Data are expressed as mean ±SD. * *p* < .05, ** *p* < .01 *t*‐test [Color figure can be viewed at wileyonlinelibrary.com]

To evaluate the efficiency of *in vivo* transduction, we performed qRT‐PCR and Western blot analysis at 48 hr postinfection. We have previously demonstrated that it is possible to detect changes in dietary behavior at this time point (Uranga et al., 2017). Rats were cannulated five days before the adenovirus injection into the 3V, and the hypothalamic tissues were collected after 48 hr, following the protocol shown in Figure 1a. As shown in Figure 1b, GLUT2 mRNA expression was reduced significantly by 75% compared with the control group. We also evaluated whether the expression of glucokinase (GK) and another glucose transporter expressed by glial cells, GLUT1, were altered by Ad‐shGLUT2 transduction. No significant differences in the expression levels of GK and GLUT1 were observed between the controls and experimental animals (Figure 1b–d).

Furthermore, we quantified the effect of Ad‐shGLUT2 transduction on the protein levels in total protein extracts of hypothalamic samples at 48 hr post‐transduction using β‐actin as a loading control. Comparison of protein extracts obtained from animals treated with control adenovirus (Figure 1e, lane 1) versus inhibitor adenovirus (Figure 1e, lane 2) indicated an effective reduction in GLUT2 levels compared with Ad‐shβgal. Overall, GLUT2 expression was reduced by 60%; however, we observed no change in the expression of GK or GLUT1 in the same samples (Figure 1f–h), indicating no compensatory effects in these proteins.

### 3.3 Adenoviral transduction specificity upon injection into the basal 3V

**Figure 2:**
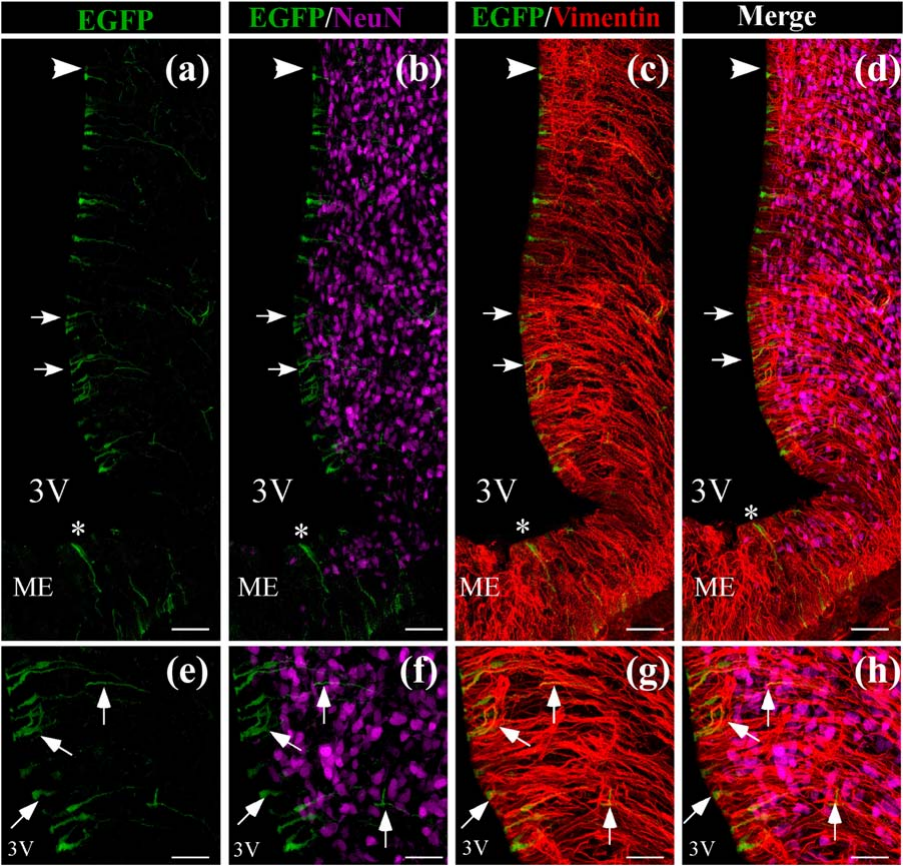
Co‐distribution of EGFP in the basal hypothalamus with neuronal and glial markers. (a‐l) Frontal sections of the hypothalamus (40 µm) were analyzed using the neuronal marker, anti‐NeuN (magenta), tanycyte marker, anti‐vimentin (red), and EGFP fluorescence (green) in cells transduced with Ad‐shGLUT2 at 48 hr postinjection. (a‐d) Low magnification for EGFP, NeuN (b and d), and vimentin (c and d). (e‐h) High magnification images of EGFP in β1‐tanycytes, NeuN (f and h) and vimentin (g and h), EGFP expression is detected in cells lining the 3V and co‐localizing with vimentin (g, arrows). Co‐localization between EGFP and NeuN (b, e, j) was not detected. i‐l: More detailed images shown in g‐h. Scale bars: a‐d: 150 µm, e‐h: 50 µm, and i‐l: 30 µm [Color figure can be viewed at wileyonlinelibrary.com]

**Figure 3:**
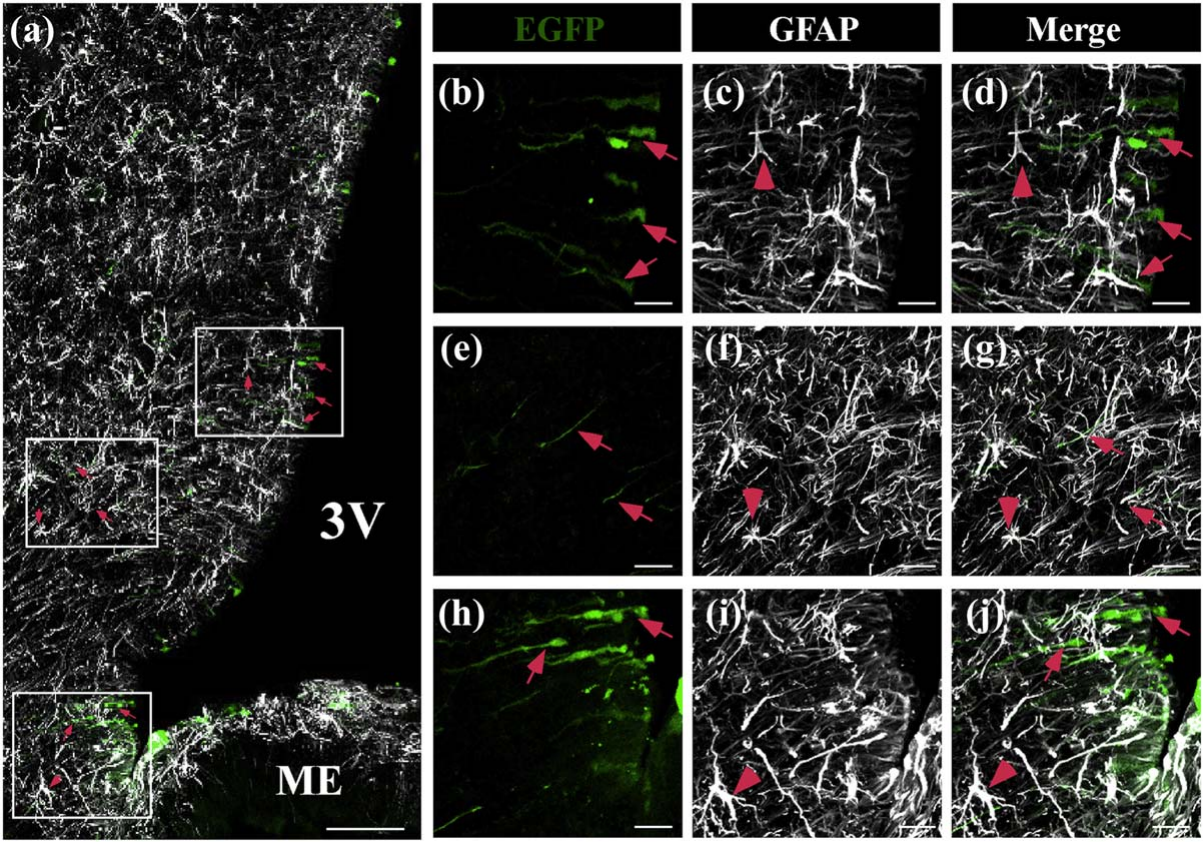
Co‐distribution of EGFP and GFAP in the basal hypothalamus. (a‐j) Frontal sections of the hypothalamus (40 µm) were analyzed using anti‐GFAP (white) and EGFP fluorescence (green) in cells transduced with Ad‐shGLUT2 at 48 hr postinjection. (a) Low magnification showing that EGFP is detected in the 3V wall and in the processes of tanycytes (red arrows). (b–j) High magnification images of the frames shown in a. Colocalization of GFAP with EGFP was not detected in astrocytes (red arrows heads). 3V: Third ventricle; ME: median eminence. Scale bars: a: 150 µm and b‐j: 50 µm [Color figure can be viewed at wileyonlinelibrary.com]

We have previously shown that 3V injection of an adenovirus with the same capsid transduces mainly tanycytes (Elizondo‐Vega et al., 2016). To corroborate that this also occurs with Ad‐shGLUT2, we used spectral confocal microscopy to evaluate EGFP expression (green), the tanycyte marker, anti‐vimentin (red), and the adult neuronal marker, NeuN (magenta) (Figure 2a–l). Consistent with previous reports, EGFP expression was detected in the wall of ventricle. The distribution of EFGP labeling together with the labeling for the intermediate filament protein, vimentin, indicates that the adenovirus transduces ependymal cells as well as α‐ and β‐tanycytes (Figure 2a–b, arrows). EGFP was also detected in some cells covering the base of the 3V and contact the ME, corresponding to β2‐tanycytes (Figure 2a, asterisk). The EGFP signal overlapped that of vimentin (Figure 2a,c,d), which was most evident at higher magnification (Figure 2g–h,k–l, arrows). However, the EGFP signal did not overlap that of Neu‐N (Figure 3b), which was most evident at higher magnification (Figure 2f,j), suggesting the absence of expression in neurons. The tropism for glial cells exhibited by the adenovirus prompted us to examine whether other glial cells besides tanycytes were transduced. As show in Figure 3a–g, the EGFP signal was detected in ependymal cells (Figure 3a,b,d, red arrows); however, it was absent in GFAP‐positive subependymal astrocytes (Figure 3c,f,i, red arrowheads). Using confocal microscopy, an assessment on the wall of the 3V was performed, and intense EGFP fluorescence was detected in ependymocytes and in tanycytes although to a lesser extent (Supporting Information Figure S2C–E), which could be explained by EGFP dilution in tanycytes (Supporting Information Figure S2A–E, arrows).

**Figure 4:**
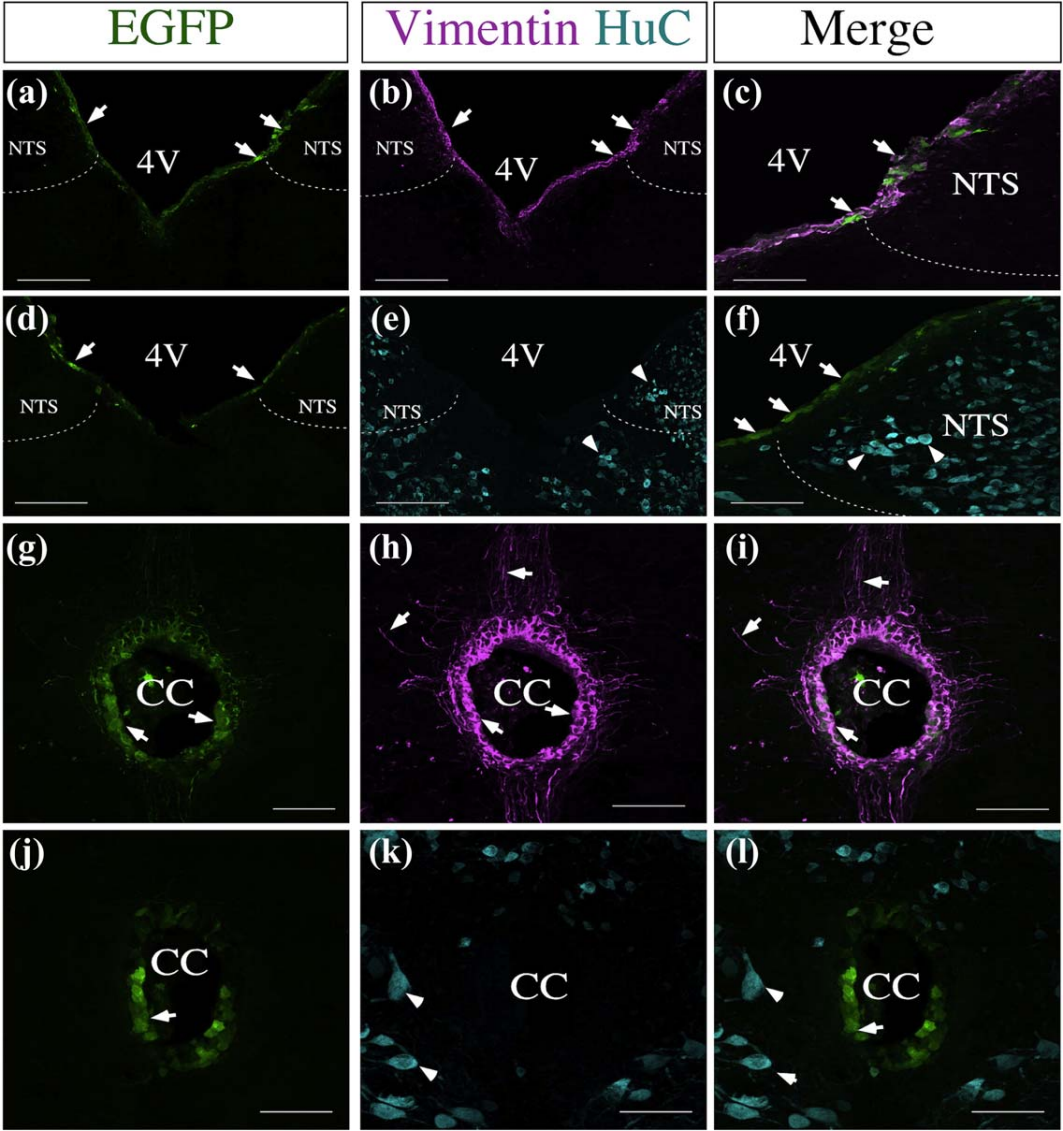
Adenovirus does not transduce brainstem neurons. (a‐l) Frontal sections of the 4V and CC (40 µm) were analyzed using the tanycyte marker, anti‐vimentin (magenta), neuronal marker, anti‐HuC (cian), and EGFP fluorescence (green) in cells transduced with Ad‐shGLUT2 at 48 hr postinjection. (a‐f) EGFP fluorescence was detected in the wall of the 4V (a and d; c and f). (b) Vimentin immunoreactivity. (c) Arrows show co‐localization between EGFP and vimentin. (d) EGFP fluorescence. (e) HuC immunoreactivity. (f) Arrowheads show the absence of co‐localization between EGFP and HuC. (g) Arrows show EGFP fluorescence in the CC. (g‐h) EGFP fluorescence in the CC. (h) Vimentin immunoreactivity. (I) Arrows show co‐localization between EGFP and vimentin. (j) EGFP fluorescence. (k) HuC immunoreactivity. (l) Arrowheads show the absence of co‐localization between EGFP and HuC. 4V: Fourth ventricle, NTS: Nucleus of the solitary tract, CC: Central cannel. Scale bar a‐f: 100 µm; G‐L: 50 µm [Color figure can be viewed at wileyonlinelibrary.com]

Because GLUT2 has been localized in the brainstem of adult mice, specifically in neurons that stimulate feeding of the NTS, we wondered whether the 3V injections could reach the NST and affect GLUT2 expression in this region. As shown in Figure 4a–c, the EGFP signal was detected at the 4V, but only in some ependymal cells. Importantly, no signal was detected in NTS neurons (Figure 4d–f). Similarly, at the central channel (CC) level, the EGFP signal was detected only in some ependymal cells (Figure 4g–i), but not in NTS neurons (Figure 4k–l).

### 3.4 GLUT2 inhibition alters the expression of orexigenic and anorexigenic neuropeptides

**Figure 5:**
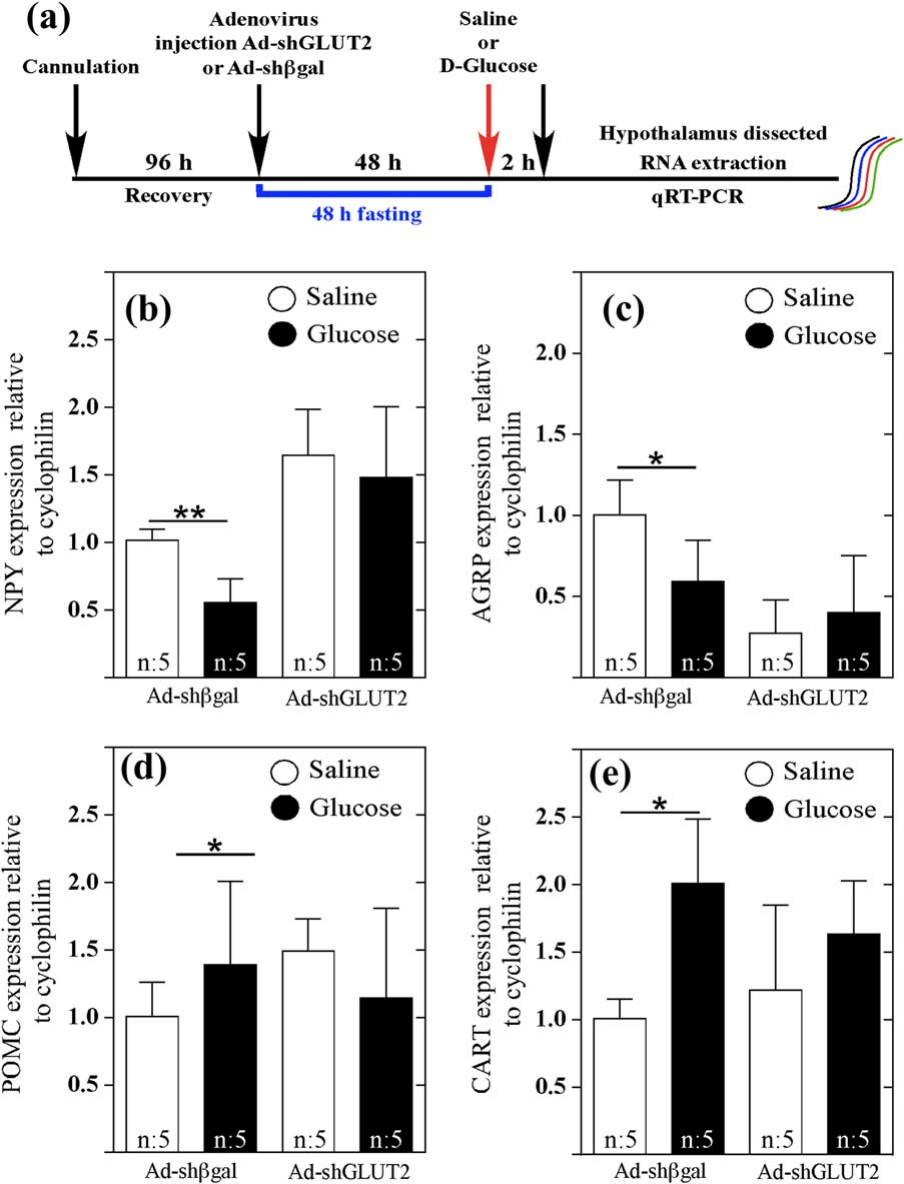
Loss of response to icv glucose injection following GLUT2 knockdown in tanycytes. (a) Experimental protocol used to analyze neuropeptide expression. Analysis of NPY (b), AGRP (c), POMC (d), and CART (e) mRNA expression by qRT‐PCR. Ad‐shβgal or Ad‐GLUT2 animals received an icv injection of aCSF (white bars) or 50 mM glucose (black bars). Total RNA was obtained 2 hr post treatment. Ad‐GLUT2 animals did not respond to icv glucose injection as measured by changes in orexigenic and anorexigenic neuropeptides. Statistical analyses were performed using nonparametric *t*‐tests. * *p* < .05; ** *p* < .01 [Color figure can be viewed at wileyonlinelibrary.com]

**Figure 6:**
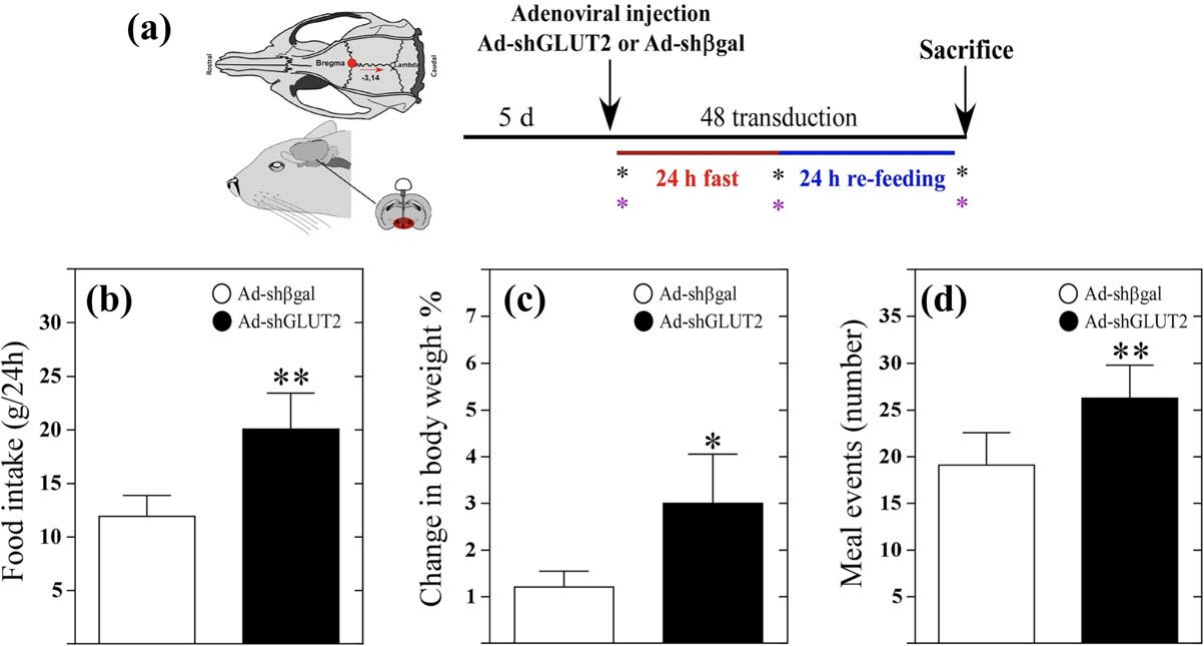
GLUT2 knockdown rats have increased food intake, body weight, and meal events. (a) Experimental protocol. Asterisks show measurements of food intake and body weight at the beginning and at the end of each stage of the fasting/refeeding cycle. (b) Food intake (g/24 hr) at 24 hr after feeding by rats transduced with Ad‐shβgal (white bars) or Ad‐shGLUT2 (black bars). (c) Change in body weight at 24 hr after feeding by rats transduced with Ad‐shβgal (white bars) or Ad‐shGLUT2 (black bars). (d) Total meal events (number) at 24 hr after feeding by rats transduced with Ad‐shβgal (white bars) or Ad‐shGLUT2 (black bars). Statistical analyses were performed using nonparametric *t*‐tests. * *p* < .05; ** *p* < .01 [Color figure can be viewed at wileyonlinelibrary.com]

Previous studies have shown that icv glucose injection produces changes in the expression of neuropeptides that signal satiety and hunger (Elizondo‐Vega et al., 2016). Neuropeptide expression was measured as shown in Figure 5a. After glucose injection, control rats showed a significant reduction in the expression of the orexigenic neuropeptides, NPY (Figure 5b, black bar) and AgRP (Figure 5c, black bar) and a significant increase in the expression of the anorexigenic neuropeptide, POMC (Figure 5d, black bar), and CART (Figure 5e, black bar). However, GLUT2 inhibition produced a loss of response to icv glucose injection as demonstrated by the expression levels of NPY (Figure 5, black bar) and AgRP (Figure 6c, black bar) that were similar to those observed with the saline treatment (Figure 5b,c, white bars). Similarly, no changes in anorexigenic neuropeptide levels were detected after icv glucose injection (Figure 5d,e, black bars), compared with the basal levels observed following saline treatment (Figure 5d,e, white bars). Taken together, our results show that GLUT2 inhibition impairs both orexigenic and anorexigenic neuropeptide responsiveness to increased glycorrhachia, possibly resulting in hunger/satiety signal dysregulation.

### 3.5 GLUT2 expression by tanycytes is necessary for food intake control

To evaluate the impact of GLUT2 inhibition on eating behavior, we measured food intake and body weight (Figure 6a). GLUT2 inhibition resulted in a significant increase in food intake compared with control rats (Figure 6b). In addition, body weight was increased in Ad‐shGLUT2‐injected rats compared with animals injected with Ad‐shβgal (Figure 6c). Total meal events, defined as the number of times that a feeding event occurs over 24 hr, was significantly larger in GLUT2‐knockdown rats compared with the control group (Figure 6d).

**Figure 7:**
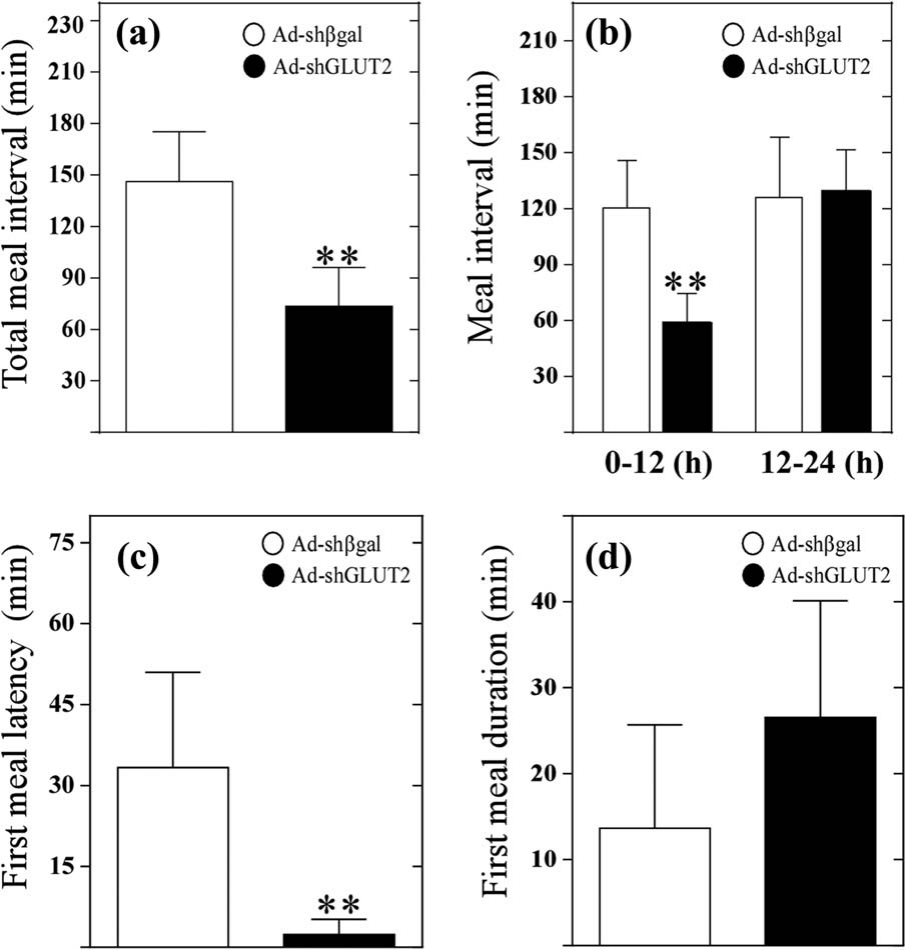
GLUT2 knockdown rats have decreased satiety. (a) Meal intervals (min) at 24 hr after feeding in rats transduced with Ad‐shβgal (white bars) or Ad‐shGLUT2 (black bars). (b) Meal interval duration at 0–12 hr and 12–24 hr in the fasting/refeeding cycle in rats transduced with Ad‐shβgal (white bars) or Ad‐shGLUT2 (black bars). (c) Latency to the first meal in at 24 hr after feeding in rats transduced with Ad‐shβgal (white bars) or Ad‐shGLUT2 (black bars) (d) Duration of the first meal at 24 hr after feeding in rats transduced with Ad‐shβgal (white bars) or Ad‐shGLUT2 (black bars). Statistical analyses were performed using nonparametric *t*‐tests. * *p* < .05; ** *p* < .01

To further explore whether these results are related to altered satiety, we analyzed the duration of meal intervals and duration of first meal. The number of inter‐meal intervals increased (data not shown), and the duration of inter‐meal intervals decreased in the Adsh‐GLUT2 group compared with the control group (Figure 7a). A more detailed analysis revealed that during the first 12 hr, GLUT2‐knockdown rats showed significant differences than observed for the control; however, these differences were lost in the following 12 hr (Figure 7b). These results are in agreement with the observed increase in meal events (Figure 6c). Moreover, GLUT2‐knockdown rats showed a minor latency in the first meal (Figure 7c) and a significantly increased duration of the first meal (Figure 7d), indicating that GLUT2‐knockdown rats were hungrier than control animals and needed more time to reach satiation. Because animals injected with Adsh‐GLUT2 showed a decrease in the duration of inter‐meal intervals our results suggest that GLUT2 inhibition impairs the mechanisms that cause satiety. In contrast, they take longer to achieve satiation, which correlates with the observed increase in body weight.

**Figure 8:**
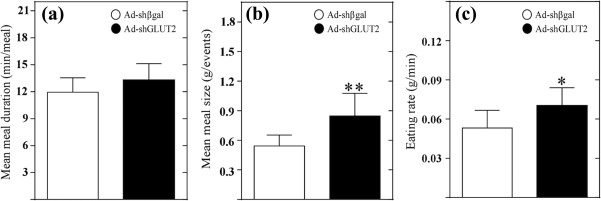
GLUT2 knockdown rats feed more rapidly than control rats. (a) Mean duration of meal events (min/number of events) over 24 hr after feeding in rats transduced with Ad‐shßgal (white bars) or Ad‐shGLUT2 (black bars). (b) Mean meal size (g/number of events) over 24 hr after feeding in rats transduced with the Ad‐shßgal (white bars) or Ad‐shGLUT2 (black bars). (c) Eating rate (g/min) over 24 hr after feeding in rats transduced with the Ad‐shβgal (white bars) or Ad‐shGLUT2 (black bars). Statistical analyses were performed using nonparametric *t*‐tests. * *p* < .005; ** *p* < .001

We also calculated other parameters of feeding behavior, including eating rate, meal duration, and meal size, as mean values for the whole cycle without making a distinction between phases (Figure 8a–c). Analysis of the mean meal duration did not show significant differences (Figure 8a). The average meal size, estimated as the total amount of food consumed in the number of total events, showed that Ad‐shGLUT2 rats consumed a significantly greater average meal size (Figure 8b). Similarly, the eating rate, estimated as the total amount of food consumed in total meal duration, showed that Ad‐shGLUT2 rats had a higher eating rate (Figure 8c), which correlated with the highly significant increase in food intake (Figure 7b).

## 4 DISCUSSION

Here, we show that GLUT2 inhibition in tanycytes disrupted the hypothalamic glucosensing mechanism that controls feeding behavior. Inhibition of GLUT2 expression resulted in increased food intake and a gain in body weight in rats. Our results are in agreement with a previous report using a GLUT2 knockout mouse model, where daily food intake was significantly increased (Bady et al., 2006). Importantly, our results indicate that tanycyte expression of GLUT2 has a role in food intake regulation.

The adenovirus construct we used was very efficient in reducing the mRNA and protein expression of GLUT2 in tanycyte cultures and in the hypothalamus *in vivo* without inducing compensatory effects in the expression of GK or GLUT1, which are also present in tanycytes. Previously, we have characterized GLUT2 kinetic properties by evaluating glucose transport in tanycytes, showing GLUT1 and GLUT2 contributions. At 20 mM glucose, the relative contribution in transport was 30% and 70% for both transporters, respectively (Garcia et al., 2003). Here, tanycyte cultures transduced with AdshGlut2 showed a significant reduction of the incorporation of glucose, indicating loss of function. Our feeding behavior results support the view that the adenovirus carrying shGLUT2 exerts an effective inhibition *in vivo*, and Western blot analysis confirmed that the protein was significantly reduced. Unfortunately, we were not able to evaluate intracellular GLUT2 expression by immunohistochemistry; therefore, it is not known whether possible changes in GLUT2 localization mediated by the transduction contributed to the loss of function. Our present results and previous findings (Elizondo‐Vega et al., 2016) indicate that injection of the adenovirus in the basal 3V results in preferential transduction in ependymal cells and tanycytes but not in neurons or astrocytes. Nevertheless, other authors have reported that serotype 5 adenoviruses exhibit a glial tropism (Cazzin et al., 2010; Chen et al., 2009). Thus, it is possible that the ependymal cells behave as a functional barrier to the adenovirus, preventing its entry into the subependymal zone, which is rich in astrocytes, and the parenchyma where neuroendocrine neurons are located. In contrast, using the same strategy (infection time and virus serotype), we have previously demonstrated that GK inhibitions in tanycytes increased food intake, meal duration, frequency of eating events and the cumulative eating time, whereas the intervals between meals were decreased, suggesting a decrease in satiety (Uranga et al., 2017). The present results indicate that inhibition of GLUT2 has an effect similar to inhibition of GK *in vivo*, suggesting that the mechanism of glucose detection requires both proteins, which supports the mechanism of indirect sensing involving tanycytes. In agreement with an indirect detection of glucose by AN neurons, rats with MCT1 knockdown lose their normal response to icv glucose and have increased food intake (Elizondo‐Vega et al., 2016).

**Figure 9:**
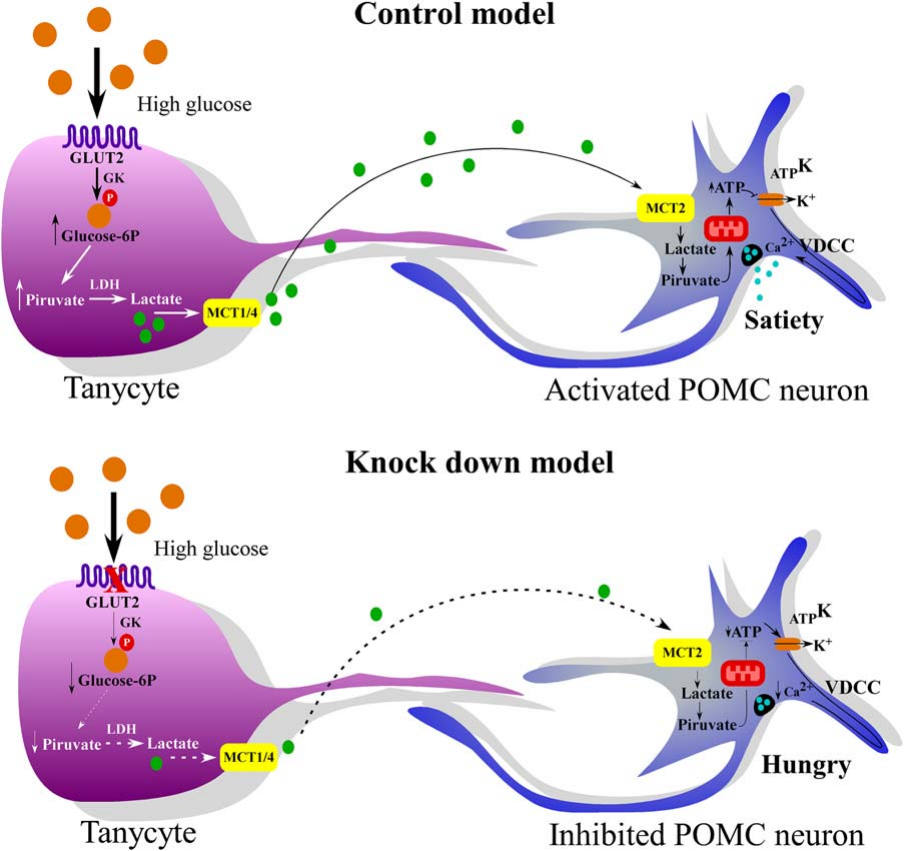
Mechanism proposed for the participation of GLUT2 expression in tanycytes to regulate the activity of anorexigenic neurons. In high glucose, tanycytes incorporate glucose through GLUT2, which is efficiently phosphorylated by the enzyme, GK. Tanycytes generate lactate, which is released through MCT1, to neighboring neurons. POMC neurons take lactate through MCT2. Lactate directs the release of POMC through ATP production. In high glucose and when GLUT2 has been partially inhibited, not enough lactate is generated by tanycytes to be transferred to neurons to activate neuronal ATP production by decreasing the release of anorexigenic neuropeptides and satiety. GK, glucokinase; MCT, monocarboxylate transporter; LDH, Lactate dehydrogenase; _ATP_K; ATP‐sensitive potassium channel; VDCCs, Voltage‐dependent calcium channel [Color figure can be viewed at wileyonlinelibrary.com]

Previously, it has been shown that tanycytes respond rapidly (minor to 2 min) to glucose by generating calcium waves that *in vitro* we shown are partly dependent on the glucose transporter, GLUT2 and depending of glycolysis and not by oxidative phosphorylation, showing the these cells have a high glycolytic activity (Benford et al., 2017; Frayling, Britton, & Dale, 2011; Orellana et al., 2012). The signals that originate by increased glucose can mediate cellular responses that could control the activity of orexigenic and anorexigenic neurons. It has been proposed that metabolic coupling between glia and hypothalamic neurons is carried out by lactate (Ainscow, Mirshamsi, Tang, Ashford, & Rutter, 2002; Thorens, 2012). For this reason and taking into account that tanycytes release lactate (Cortes‐Campos et al., 2011), we proposed that the lactate released by tanycytes in response to glucose is transferred to POMC neurons producing an increase in intracellular ATP, in a mechanism that is similar to that reported for pancreatic β‐cells (Meda & Schuit, 2013). In this mechanism, the increase in ATP induces the closure of K_ATP_ channels and subsequent membrane depolarization opening of voltage‐dependent calcium channels and release of αMSH, derived from the POMC to produce satiety. The partial inhibition of GLUT2 in tanycytes would result in lower glucose uptake, reduced lactate genesis, and interrupted POMC neuronal activity, with a subsequent decrease in intervals between meals, reflecting a decrease in satiety, which could explain the increased food intake and body weight in GLUT knockdown animals (Figure 9).

Interestingly, a transgenic line that lack GLUT2 (RIPGlut1;GLUT2^−/−^) shows increased daily food intake with abnormal feeding initiation and termination following a fasting period (Bady et al., 2006). Likewise, inhibiting GLUT2 expression resulted in a similar pattern of response in neuropeptide expression and food intake; however, the rats also showed an increase in body weight. This discrepancy could be due to differences in the species used in these experiments. Also, in our experiments, the reduction in GLUT2 expression was limited to ependymal cells and tanycytes, while in the transgenic line, expression was also affected in neurons sensitive to hypoglycemia located in the brainstem (Tarussio et al., 2014) or neurons in the thalamus involved in carbohydrate preference (Labouebe et al., 2016). We also showed that suppression of GLUT2 expression in tanycytes leads to the loss in response to increased icv glucose, supporting a role for indirect control of glucose over the expression of neuropeptides that control hunger and satiety.

Interestingly, tanycytes have been postulated to function as neuromodulating cells, since they can regulate the availability of peripheral hormones, such as leptin (Balland & Prevot, 2014) and ghrelin (Collden et al., 2015), from peripheral tissues to neurons of the AN. This is because tanycytes link the ventricular and vascular compartments, forming a blood/CSF interface in the tubal region of the hypothalamus (Langlet et al., 2013). β2‐tanycytes have long processes that extend to the perivascular space of the capillary network of the ME to reach the fenestrated capillary network. Thus, it is feasible to propose that β2‐tanycytes concentrate glucose in the CSF of the infundibular recess in order to be transferred to β1‐ and α‐tanycytes, the populations the responsible for communicating glucose concentration to neuroendocrine AN neurons. Although it is known that the concentration of glucose in CSF increases proportionally to the blood (Steffens, Scheurink, Luiten, & Bohus, 1988), the changes of glucose concentration in the CSF of the 3V changes during the feeding fasting transition is unknown. We have previously shown that hyperglycemia can be induced by ip injection of glucose in the CSF with an increase of up to 10 mM glucose occurring (Salgado et al., 2014). These antecedents suggest that tanycytes are responsible for detecting changes in glucose concentration from the CSF and generating a signal that allows the generation of a response by the adjacent neuron, forming a circuit that involves both cell types in a coupled manner. On the other hand increased lipid droplet content in tanycytes has been observed after a prolonged high fat diet (Hofmann et al., 2017), which is in agreement with a role of tanycytes in hypothalamic lipid sensing (Levin, Magnan, Dunn‐Meynell, & Le Foll, 2011). Thus, due to their localization in the hypothalamus, tanycytes are in a privileged position to detect hormonal, nutritional and mitogenic signals released by peripheral organs or present in the CSF. Recent studies reveal that in response to nutritional signals, tanycytes are capable of differentiating into anorexigenic neurons (Gouaze et al., 2013; Recabal et al., 2017), which strongly supports the notion that these cells are involved in the control of feeding behavior.

## CONFLICT OF INTEREST

The authors declare that they have no competing interests.

## AUTHOR CONTRIBUTIONS

The experiments were performed at the Department of Cell Biology at the University of Concepcion. MAG‐R, EU, and FS conceived the experiments; MAG‐R, MJB, and RE‐V, AR designed the experiments; MJB, PLL, KE‐A, RE‐V, PO, AR, and MS performed the experiments; MJB, PLL, RE‐V, PO, and MS analyzed the data; MAG‐R, RCA, FS, and EU contributed reagents/materials/analysis tools; MAG‐R and MJB wrote the article; and FS, RCA, and EU critically revised the manuscript. All authors have approved the final version of the manuscript and agree to be accountable for all aspects of the work in ensuring that questions related to the accuracy or integrity of any part of the work are appropriately investigated and resolved. All persons designated as authors qualify for authorship, and all those who qualify for authorship are listed.

## Supporting information

Additional Supporting Information may be found online in the supporting information tab for this article.

Supporting Information
