# Supplementary material for: Glial hypothalamic inhibition of GLUT2 expression alters satiety, impacting eating behavior
Source: Glia. 2017 Nov 27;66(3):592–605. doi: 10.1002/glia.23267 (PMC5814884; doi:10.1002/glia.23267)
Supplement: Supplementary file 1 — Supporting Information [file GLIA-66-592-s001.doc]

Glial hypothalamic inhibition of GLUT2 expression alters satiety, impacting eating behavior

María J. Barahona^1^, Paula Llanos^1^, Antonia Recabal^1^, Kathleen Escobar^1^, Roberto Elizondo-Vega^1,5^, Magdiel Salgado^1^, Patricio Ordenes^1^, Elena Uribe^2^, Fernando J Sepúlveda^2,3^, Ricardo C Araneda^4^ and María A. García-Robles^1^

^1^Laboratorio de Biología Celular, Departamento de Biología Celular, Facultad de Ciencias Biológicas, Universidad de Concepción, Concepción, Chile; ^2^Departamento de Bioquímica y Biología Molecular, Universidad de Concepción, Chile; ^3^Departamento de Ciencias Biológica Universidad Andrés Bello, Concepción Chile; ^4^Department of Biology, University of Maryland, College Park MD, USA. ^5^Laboratorio de Inmunología Celular y Molecular, Centro de Investigación Biomédica, Facultad de Medicina, Universidad de los Andes, Santiago, Chile.

**Corresponding author:** María A. García-Robles Laboratorio de Biología Celular, Departamento de Biología Celular, Facultad de Ciencias Biológicas, Universidad de Concepción, Concepción, Chile.

**
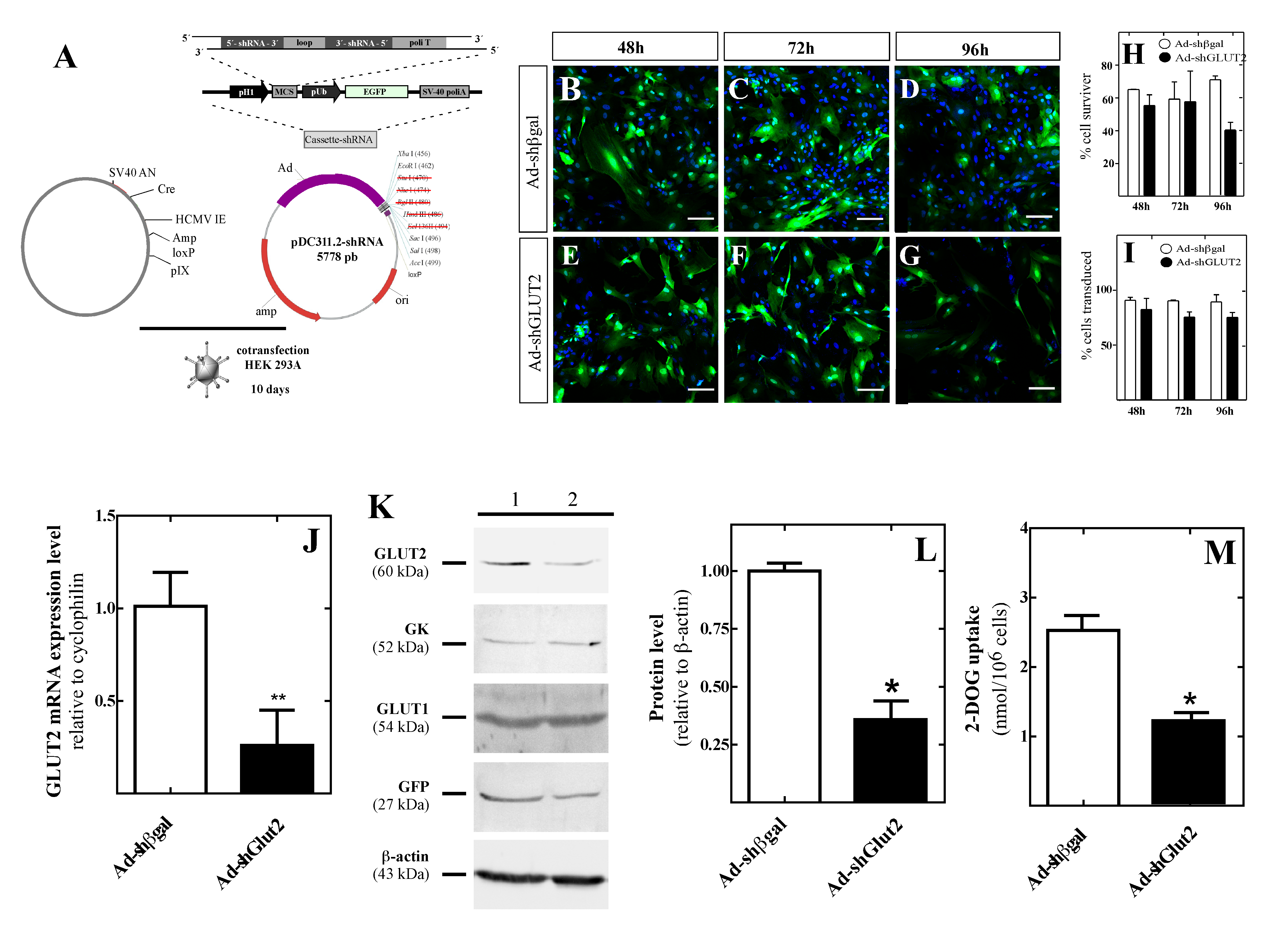
**

**Supplementary Figure 1: Generation of adenoviruses and transduction in primary cultures of hypothalamic tanycytes.**

**A:** Experimental protocol that shows the construction of the adenoviral shuttle vector. **B-D:** Ad-shßgal-transduced tanycytes. **E-G:** Ad-shGLUT2-transduced tanycytes. **H:** Percentage of surviving tanycytes following transduction with Ad-shβgal (white bars) and Ad-shGLUT2 (black bars) at 48 h. **I:** Quantification of EGFP expression normalized to total tanycytes following transduction with Ad-shβGal (white bars) or Ad-shGLUT2 (black bars). **J:** Analysis of GLUT2 mRNA expression in tanycytes transduced for 48 h with Ad-shßgal or Ad-shGLUT2 by qRT-PCR following normalization with cyclophilin. **K:** Western blot analysis of protein extracts isolated from tanycytes transduced for 48 h with Ad-shβgal (lane 1) or Ad-shGLUT2 (lane 2). **L:** Densitometric analysis of GLUT2 relative to β-actin in Ad-shβgal- and Ad-shGLUT2-transduced cells. M: Uptake of 20 mM 2-DOG. Experiments were performed at room temperature. Results are representative of three independent experiments performed in triplicate. Data are expressed as mean +-SD. * *p*<0.05, t-test. Scale bars: 100 μm.


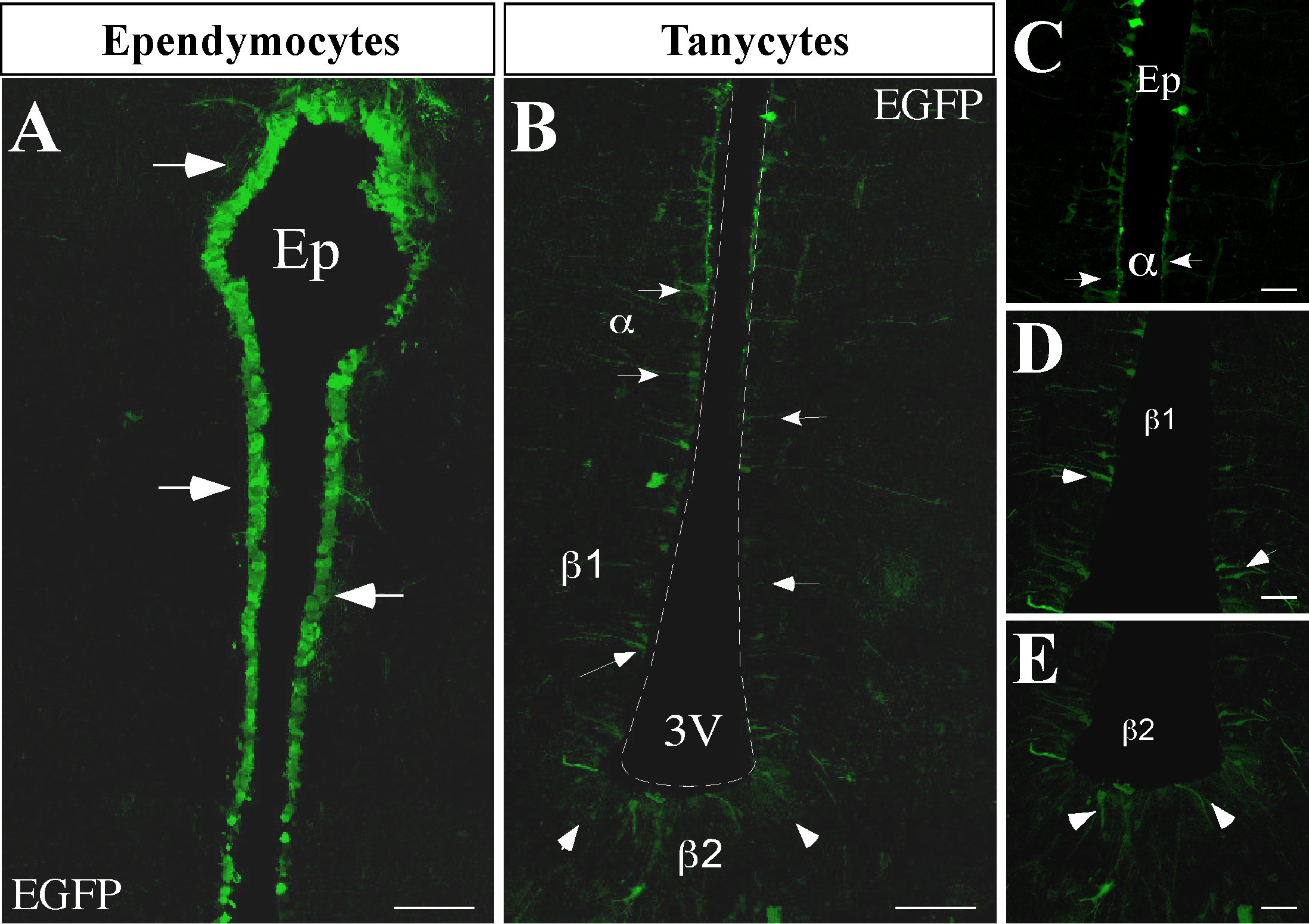


**Supplementary Figure 2: Adenovirus specifically transduces ependymocytes and tanycytes of the third ventricle.**

**A-B:** Frontal sections of the hypothalamus (40 µm) in which EGFP fluorescence (green) is shown in cells transduced with Ad-shβgal at 48 h post-injection. **A:** Low magnification showing that EGFP is detected in ependymocytes located in dorsal third ventricle wall. High magnification image of the frames shown in B. EGFP fluorescence is observed in α- **(C),** β1- **(D),** and β2-tanycytes **(E).** Ep: Ependymocytes. Scale bar: A-B:100 µm; C-E:50 µm.
